# Additive manufacturing - an antidote to bottlenecks in tissue engineering and regenerative medicine

**DOI:** 10.3389/fbioe.2026.1929172

**Published:** 2026-09-08

**Authors:** Yifan Li, Fabian Tribukait-Riemenschneider, Yawei Gu, V. Prasad Shastri

**Affiliations:** 1 Institute of Macromolecular Chemistry, University of Freiburg, Freiburg, Germany; 2 Department of Anatomy, Engineering Research Center for Organ Intelligent Biological Manufacturing of Chongqing, Key Lab for Biomechanics and Tissue Engineering of Chongqing, Army Medical University (Third Military Medical University), Chongqing, China; 3 Engineering Research Center of Tissue and Organ Regeneration and Manufacturing, Ministry of Education, Chongqing, China; 4 BIOSS – Centre for Biological Signalling Studies, Freiburg, Germany

**Keywords:** biofabrication, biomaterials, bioprinting, regenerative engineering, translational bioengineering

## Abstract

Tissue engineering and regenerative medicine (TERM) has advanced by leveraging innovations in biomaterials, cell biology, and drug delivery, but its potential remains to be fully realized due to limitations in manufacturing complex cellular constructs. The complexity of cellular and extracellular matrix (ECM) organization poses a formidable challenge to replicate using conventional fabrication methods. Additive manufacturing (AM) enables the translation of design intent into controlled three-dimensional (3D) constructs with defined geometry, internal architecture, and spatially organized components. Beyond merely fabricating complex structures, its value lies in addressing structural, spatial, reproducibility, personalization, and translational limitations. However, printability does not guarantee biological function. By exploring AM of soft matter (hydrogels) as a practical manufacturing framework for TERM, this review first identifies the core manufacturing bottlenecks in classical TERM (Section 2), then evaluates how AM addresses structural fidelity, spatial patterning, digital reproducibility, and personalization (Section 3). Using osteochondral units, skin, and vascular grafts as representative testbeds (Section 4), we critically analyze current limitations—including bioink tradeoffs, resolution-scale constraints, and regulatory barriers (Section 5)—and outline the functional validation, standardization, and digital-AI integration needed for clinical implementation (Section 6). The review concludes that AM’s translational impact will depend less on geometric complexity than on demonstrating tissue maturation, host integration, and regulatory compliance within viable clinical workflows.

## 1 TERM still faces a manufacturing problem

Tissue engineering (TE) and regenerative medicine (TERM) aims to restore tissue structure and function after injury, degeneration, congenital defects, or disease. Clinical success in TERM is defined by tissue-specific functional performance, not construct specifications alone, thereby establishing the criteria against which scientific progress must be measured. Such a function-based assessment is exemplified in some recent studies. Bioprinted skin models have been evaluated through epidermal and dermal maturation, barrier-related structure, and immune responsiveness. Microfluidic bioprinted hydrogel vascular conduits have been assessed by vascular marker expression, barrier function, and vasomotor response. Spatiotemporally defined growth factor patterns in bioprinted osteochondral constructs have been examined through cartilage and bone-related differentiation. Personalized thick and perfusable cardiac patches have been evaluated through patient-specific geometry, vascular organization, and contractile tissue formation (Bhar et al., 2024; Wang D. et al., 2022; Freeman et al., 2020; Sun et al., 2020; Noor et al., 2019). These five cases show that TERM is not judged only by whether living cells or biomaterials can be combined, but by whether engineered systems can meet tissue-specific functional demands in a clinically meaningful environment.

Despite substantial progress in biomaterials, stem cell biology, growth factor delivery systems, and bioreactors and culture systems, many TERM strategies remain difficult to translate. A fundamental barrier is that effective regeneration represents not solely a biological challenge, but equally a manufacturing one. For instance, fabricating thick tissues requires integrated, perfusable vascular channels to sustain high cell densities (Skylar-Scott et al., 2019). Similarly, engineering cardiac tissues demands manufacturing scaffolds capable of recapitulating the complex collagenous architecture of the aorta, coronary artery, valves, and chambers (Lee et al., 2019). Consequently, the successful translation of TERM strategies hinges on achieving predictable and controllable physical properties (e.g., stiffness, porosity), in conjunction with biological functionality.

Engineered tissues require coordinated control over construct geometry, pore structure, mechanical properties, cell distribution, and bioactive signal localization; fabrication should preserve cell viability and material stability while supporting nutrient transport, culture, implantation, and functional evaluation under reproducible conditions–integrating these multifaceted requirements represents the foremost challenge of TERM. Spheroid-based bioprinting of high-cell-density tissues has shown that spatial organization can be defined before microtissue fusion, highlighting the need to control both the initial arrangement of tissue building blocks and their later biological assembly (Daly et al., 2021). Conventional methods such as molding, casting, freeze-drying, electrospinning, and manual cell seeding have contributed substantially to scaffold engineering for TERM applications. Variations in scaffold geometry, material distribution, and quality of raw materials complicate both inter-batch consistency and inter-study comparability, thereby hindering clinical translation. Early work using hydrocarbons as a sacrificial phase to generate macroporous polymer foams with controlled pore structure provides a historical precedent for architecture-controlled and standardized scaffold design (Shastri et al., 2000), which lends itself well to exploiting multimaterial printing in additive manufacturing (AM) of macroporous polymer foams. In regard to cell systems, high-throughput spheroid bioprinting has been exploited to address the scalability of laboratory-scale TERM approaches, further emphasizing that scalable tissue fabrication requires reproducible placement and fusion of biological building blocks (Kim et al., 2024). Additive manufacturing can help address these limitations by linking digital design with controlled material deposition, and is poised to elevate the complexity, reproducibility, and scalability of engineered cellular constructs.

The value of AM lies not simply in producing complex structures, but in translating predefined designs into constructs with defined architecture (graded mechanical and structural attributes, porosity) and spatially resolved biological information, thereby enabling scalable production through modular assembly. Through computer-aided design, imaging-based modeling, toolpath generation, and bottom-up fabrication, AM can integrate scaffold fabrication, cell placement, and presentation of bioactive signals within a single, digitalized workflow (Figure 1). The potential of AM is therefore reflected in multiscale embedded printing of engineered human tissue and organ equivalents, model-guided design of organ-scale synthetic vasculature, and high-speed embedded ink writing of anatomical-size organ constructs (Zhang et al., 2024b; Sexton et al., 2025; Hua et al., 2025). AM should not be viewed as a panacea or a universal solution for TERM, but rather as an antidote for certain challenges and bottlenecks that limit the progress of the field. While AM encompasses different techniques such as fused deposition modeling of thermoplastics, direct ink writing (extrusion printing) of soft matter, drop-on-demand printing of cell suspensions, and light-based techniques like stereolithography, this review focuses specifically on the AM of soft materials as it pertains to TERM. Here, we first identify the principal manufacturing bottlenecks in classical TERM—structural complexity, spatial control, mechanical maturation, and standardization. We then examine how AM addresses these through shape fidelity, spatial patterning, digital workflows, personalization, and integrated biofabrication. To illustrate tissue-specific opportunities, we turn to osteochondral, skin, and vascular graft testbeds. A critical evaluation follows, covering AM limitations including the printability-function gap, material constraints, resolution-scale tradeoffs, and regulatory barriers. Finally, we outline the functional validation, standardization, and digital integration required for clinical translation.

**FIGURE 1:**
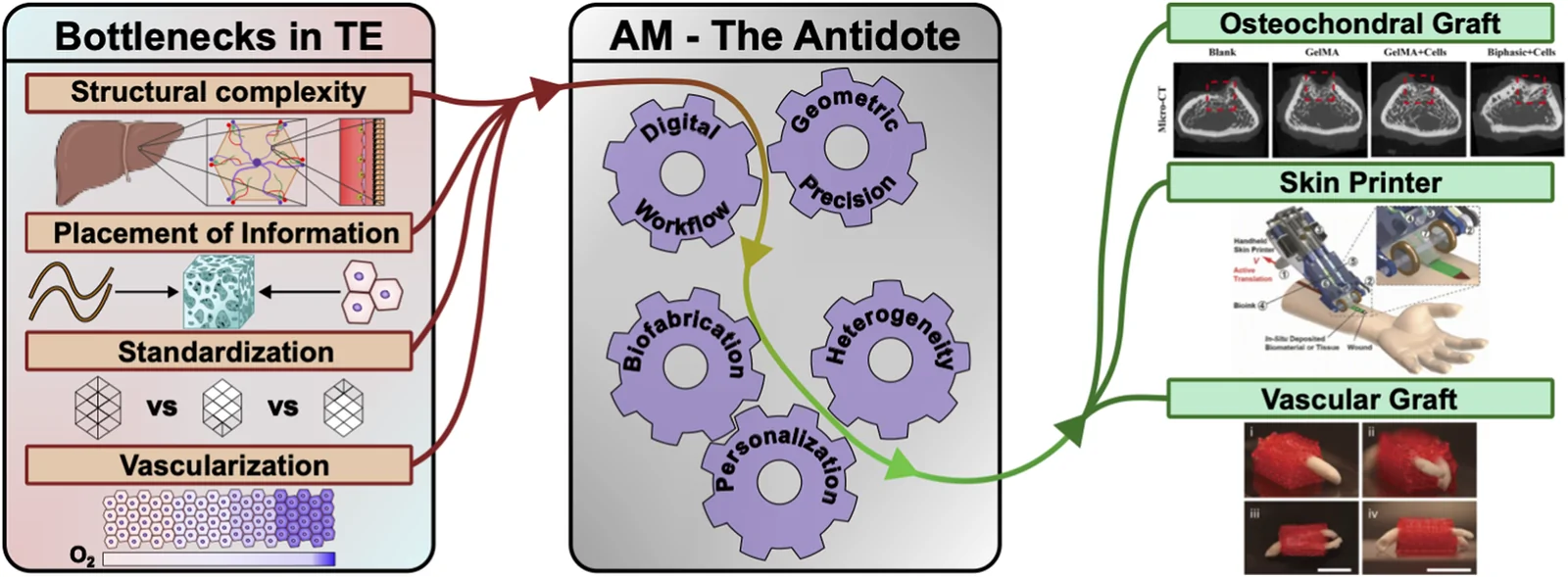
The key attributes of additive manufacturing (AM) (center box) play a role in addressing bottlenecks in conventional tissue engineering (TE) (left box). This interplay between AM and TE–leading to TE aided by AM (TEAM), has already shown potential in interface engineering of complex tissues (right box) such as biphasic, osteochondral tissue unit (Yu et al., 2024) (reproduced under creative common license), direct printing of skin-like interface (Hakimi et al., 2018) (reproduced with permission), and layered architecture of an arterial vessel (Szklanny et al., 2021) (reproduced under creative common license).

## 2 Bottlenecks in TERM

### 2.1 Structural and hierarchical complexity

A core limitation in TERM is the difficulty in reproducing the nuanced complexity of native tissues with regard to anatomical attributes, extracellular matrix (ECM) composition, mechanics, physiology, and biochemistry. In the joint, cartilage, calcified cartilage, and subchondral bone constitute a contiguous structure characterized by graded, yet distinct, compartmentalized mechanical and biological functions. Such organizational paradigms are also found in skin, blood vessels, spinal cord, and intestinal lumen. While conventional scaffolds (foams, fibers, meshes) can provide mechanical support, they are limited in their ability to replicate anisotropy and structural hierarchy within one construct. In osteochondral repair, construct geometry must align with interfacial mechanics to facilitate neocartilage integration and restore energy dissipation (Brown et al., 2021). The need for a patent interface between cartilage and subchondral bone was already recognized early on, leading to the development of cartilage-bone composites via the fusion of independently engineered (cartilage and mineralized) modules (Schaefer et al., 2000). This modular approach served as a precursor to modern AM strategies aimed at addressing tissue interfaces. This approach has also been exploited in cell-laden hydrogel bioinks (Kilian et al., 2020). These studies highlight the need to incorporate spatial hierarchy and mechanical transition during construct design.

### 2.2 Spatial control over cells, signaling, and vascularization

Another bottleneck is the limited precision in localizing cells, biomaterials, and biological signals within engineered constructs. Tissue regeneration depends not only on the presence of these components, but also on their local density, regional distribution, and temporal presentation. Random cell seeding or uniform incorporation of bioactive molecules may be insufficient when the target tissue requires zonal organization or interface formation. For interface repair, adjacent regions may require different cell phenotypes, matrix compositions, or mechanical properties. For interface repair, adjacent regions may require different cell phenotypes, matrix compositions, or mechanical properties, yet conventional fabrication methods lack the precision to position distinct cell populations and biomaterials in spatially defined zones (Qin et al., 2021). Spatiotemporally patterned growth factor printing and dual-factor gradient constructs further show that spatially organized biological signals can guide vascularization, osteogenesis, and anisotropic cartilage regeneration (Sun et al., 2020; Freeman et al., 2020). In addition to growth factor patterning, bioprinting can yield constructs with stiffness and cell adhesion to direct cell behavior via mechanobiology (Kuzucu et al., 2021), while predefined cellular organization can also be accomplished using spheroid and organoid printing and reinforces regional cell distribution as a functional design parameter (Brassard et al., 2021; Daly et al., 2021).

To engineer functional large-scale tissues, a perfusable vasculature is essential, as diffusion becomes severely limited beyond the ∼100–200 µm–the oxygen diffusion limit. Consequently, nutrients and metabolic waste must be transported via an intricate, perfusable network to achieve tissue maturation. Pre-vascularization thus represents a prerequisite for engineering thick tissues. This is particularly critical for zonated or compartmentalized organs such as the liver, where vascular flow and metabolic gradients are intrinsically linked to region-specific cellular functions. Cell-cell communication can play a vital role through juxtacrine signaling. In multicellular spheroids, mesenchymal-endothelial interactions involving mechanosensing Notch signaling have been shown to drive *de novo* vascular structure formation (Li et al., 2022). Furthermore, achieving functional vascularization in thick constructs requires hierarchical vessel networks that span from macroscopic conduits to capillary-scale structures across the full tissue thickness (Szklanny et al., 2021). Model-guided design of organ-scale synthetic vasculature further indicates that transport networks need to be designed as functional architectures rather than being added afterward (Sexton et al., 2025).

### 2.3 Mechanical attributes and functional maturation

Appropriate mechanical property profiles are essential for fabrication, handling, culture, implantation, and early physiological loading, particularly for load-bearing tissues such as cartilage and vascular grafts. However, the simultaneous optimization of mechanical stability and biological performance remains challenging. Soft, cytocompatible materials may lack structural stability, whereas mechanically robust constructs may restrict cell spreading, remodeling, or matrix deposition. Therefore, structured fabrication alone does not necessarily lead to functional maturation. Using bioprinted tough hydrogel-based vascular conduits, it has been shown that hydrogel mechanics, endothelial function, and vascular responsiveness need to be coordinated within a single construct (Wang D. et al., 2022). Through enzymatic post-print processing, high fidelity can be combined with material softening to promote cell function within gelatin methacrylate (GelMA) and hyaluronic acid methacrylate (HAMA) constructs (Wang M. et al., 2022).

### 2.4 Standardization and scale: Path to translation

Reproducibility and standardization remain the Achilles’ heel of the clinical translation of TERM. Many TE protocols depend on manual handling and are impacted by variability in cell source, batch-to-batch variation, biomaterial inconsistencies, and laboratory-specific processing conditions. AM can augment the TE workflow by driving innovation in customized labware compatible with robotic automation and liquid handling systems. In spheroid-based biofabrication, spheroid preparation is a dominant variable. In this context, a 3D-printed hanging-drop insert optimized for handling and robotics improved spheroid quality, providing upstream standardization of biological input (Butelmann et al., 2022). Inadequate standardization also creates regulatory challenges, as many emerging biomaterials and biofabricated constructs still lack well-established quality specifications, manufacturing controls, and clear approval pathways for clinical use. Flow sensor-based monitoring of extrusion bioprinting has shown that pressure-controlled and flow-controlled printing modes can produce different levels of process stability (Strauss et al., 2022). Notwithstanding these needs, standardization must not compromise therapeutic personalization. For bioprinted TERM solutions, personalization should extend beyond external contour matching. A patient-specific construct should also account for the biological payload, local tissue environment, vascular or perfusion requirements, and the expected post-printing tissue response. *In situ* skin bioprinting serves as a case in point, where autologous cell delivery can be matched to wound geometry, integrating biological payload, deposition context, and post-printing tissue response (Albanna et al., 2019).

## 3 AM as a path to overcoming limitations in classical TE

AM provides a practical route to circumvent key manufacturing barriers that have impeded the clinical translation of TERM. Its main contribution extends beyond the fabrication of complex shapes to the faithful translation of prescribed designs into physical constructs with controlled geometry, architecture, and spatial organization. This capability is important because successful regenerative strategies dictate a close interplay between structure, material distribution, cellular positioning, and biological function. By integrating digital models, programmable deposition, and layer-by-layer fabrication, AM enables construct design prior to fabrication and post-fabrication assessment with greater consistency. Studies utilizing ECM-reinforced bioinks show how biological matrix cues can be incorporated into printable human tissue systems (De Santis et al., 2021). Furthermore, *in situ* bioprinting with bioconcrete bioink and dynamic hydrogel bioinks further demonstrates that material formulation can combine printability, stabilization, and biological compatibility (Xie et al., 2022; Hull et al., 2023).

### 3.1 Shape fidelity and perfusable architecture

One clear advantage of AM lies in its ability to achieve high structural fidelity and precise dimensional control—a prerequisite for both cell-free and cell-laden constructs. While conventional fabrication methods can yield 3D constructs, they afford limited control over material homogeneity and internal structure. In bioprinting, geometric precision is paramount when coupled with cytocompatible materials and perfusable channels to recapitulate tissue-specific functions. Hydrogels with multivascular structures show how printed geometry can be converted into perfusable architectures by combining fluid mixing and intravascular topology (Grigoryan et al., 2019). Using the Freeform Reversible Embedding of Suspended Hydrogels (FRESH) method, scaled-down collagen-based vascular structures and cardiac components have been printed with high fidelity and authenticity to their anatomical counterpart (Lee et al., 2019). Together, these studies indicate that AM improves shape control not only at the external construct boundary, but also within internal channel networks and multiscale tissue architectures.

### 3.2 Spatial patterning and heterogeneity

AM can also enable spatial control over mechanical attributes and bioactive components. Many tissues contain regional differences in matrix composition, cell phenotype, biochemical cues, and mechanical properties. Consequently, homogeneous scaffolds are often inadequate for tissues characterized by gradients, interfaces, layered organization, or intrinsic heterogeneity. With advanced printing strategies, different biomaterials, cell populations, and soluble factors can be positioned in predefined regions of the construct. In osteochondral repair, for example, a construct requires a cartilage-like region, a mineralized region, and an interfacial zone to effectively transfer load. Similarly, skin or vascular tissues demand regional organization to reproduce compartment-specific functions. Multicellular osteochondral bioprinting has been used to spatially place chondrocyte-laden and MSC-laden bioinks into cartilage-like and subchondral bone-like regions, creating a heterogeneous construct for osteochondral repair (Qin et al., 2021). Spatially controlled growth factor release from printed HAMA-heparin hydrogels has shown that signal localization and release kinetics are important design parameters beyond static geometry (Wang et al., 2020). Beyond discrete regional assignment, additive manufacturing can reproduce the continuous gradients of native osteochondral tissue, where collagen orientation, mineral content, porosity, and stiffness transition progressively from articular surface to subchondral bone (Beeren et al., 2024). 3D-printed scaffolds using mineralized bacterial cellulose nanofibers exploit this principle to establish a mineralization gradient spanning cartilage-like and bone-like regions (Liu et al., 2025), while bilayered hydrogels with Kartogenin gradient has been shown to drive zone-specific chondrogenesis and endochondral ossification from a single cue (Wei et al., 2023).

### 3.3 Digital workflow and reproducible manufacturing

Another important advantage of AM is its digital workflow. Imaging, computer-aided design, toolpath generation, printing, and post-fabrication assessment can be integrated within a single manufacturing feedback loop. This workflow permits iterative optimization, in which construct design and material distribution can be modified based on mechanical testing, imaging, biological outcomes, or computational modeling. While this does not remove biological variability associated with primary cells or natural biomaterials, it significantly reduces process-related variability linked to manual fabrication, thereby enhancing transparency and reproducibility. For instance, projection stereolithography has been used to convert complex digital vascular topologies into perfusable hydrogel constructs with functional intravascular architectures, including fluidic mixers and valve-like structures (Grigoryan et al., 2019). More recently, model-guided vascular design has integrated rapid vascular network generation, computational fluid dynamics, and 3D bioprinting, demonstrating how digital workflows support the scalable design and fabrication of perfusable tissue constructs (Sexton et al., 2025).

### 3.4 Personalization and defect-specific fabrication

Personalization is another crucial translational advantage of AM. Patient defects differ considerably in their geometric dimensions and local environment, necessitating solutions beyond standardized scaffolds. AM can leverage patient imaging data to design constructs that precisely match anatomical geometry, which is vital for irregular bone defects, craniofacial reconstruction, and osteochondral lesions where shape congruence affects fixation, integration, or function. From a deeper structural standpoint, personalization should extend beyond external shape to encompass adjustments in internal architecture, local stiffness, degradation behavior, and biological loading tailored to clinical needs. Curvilinear bioprinting of tri-cell-laden polyurethane-gelatin hydrogels for irregular wounds showed how cell-laden bioinks can be deposited according to defect topography, supporting customized tissue fabrication beyond a “one-size-fits-all” approach (Wu et al., 2022).

### 3.5 Combination of advanced biofabrication strategies

AM serves as a versatile platform capable of unifying diverse biofabrication strategies—including 3D bioprinting, sacrificial templating, support baths, microfluidics, and bioreactor maturation—into a single cohesive workflow. Sacrificial materials can be used to create perfusion channels, while printed hydrogel phases can carry cells, matrix components, or biological signals. This integrative capacity is attractive, as no single bioink or printing method can fulfill all the requirements of a functional tissue construct. Thermogelling materials such as, gelatin can be combined with a primary bioink to complement (or improve) the property profile of bioinks (complementary bioink) to assist during printing and provide support for printed structures (Singh et al., 2019). Volumetric bioprinting has shown the capability to fabricate centimeter-scale organoid-laden hydrogel constructs while preserving hepatic metabolic functions (Bernal et al., 2022). Additionally, double interpenetrating network bioinks further illustrate that printed structures can achieve immediate stability for post-print assembly of hydrogel components, allowing AM to be combined with modular assembly strategies to build more complex multi-component tissue constructs (Tribukait-Riemenschneider and Shastri, 2026). Bioprinted structures with core-shell architecture, when combined with strategies to promote growth factor affinity to the bioink, can yield controlled release of growth factors within printed structures (Wang et al., 2020).

Overall, the role of AM in TERM should be reconceptualized as a manufacturing framework that supports controlled architecture, spatial organization, reproducibility, and personalization–effectively serving as a bridge toward precise heterogeneity. It is worth noting that AM does not solve all biological problems; cells still require a suitable microenvironment, vascular support, mechanical stimulation, and sufficient time to form mature tissue. Nevertheless, AM does provide a level of design control unattainable by conventional TE methods. Its value lies in making structure, composition, and fabrication parameters more controllable, measurable, and adaptable to specific regenerative goals. Recent advances in multiscale and high-speed embedded printing indicate that AM contributes most significantly when geometric design, material selection, spatial biology, and process monitoring are treated as interconnected facets of a unified manufacturing challenge, rather than as isolated demonstrations of printability (Zhang et al., 2024b; Hua et al., 2025). In Table 1, we have summarized the above-mentioned bottlenecks and their respective promising AM solutions.

**TABLE 1:** Classical TERM manufacturing bottlenecks and corresponding AM solutions.

Classical TE bottleneck	AM solution	Key materials/Bioinks	Representative studies
Structural hierarchy and anisotropy	Layer-by-layer graded fabrication	GelMA/AlgMA, collagen, silk-gelatin	Brown et al., 2021; Kilian et al., 2020; Zhang et al., 2024b
Spatial localization of cells and signals	Multi-material/multi-channel deposition	Cell-laden hydrogels, HAMA-heparin	Qin et al., 2021; Wang et al., 2020; Freeman et al., 2020
Vascularization of thick tissues	Sacrificial templating; embedded printing	FRESH collagen, vascular bioinks	Grigoryan et al., 2019; Lee et al., 2019; Sexton et al., 2025
Mechanical-functional maturation	Tunable/double-network bioinks	CA-collagen, GelMA/HAMA, tough hydrogels	Wang D. et al., 2022; Tribukait-Riemenschneider and Shastri, 2025
Reproducibility and standardization	Digital workflow; sensor-monitored printing	Thermogelling composites	Strauss et al., 2022; Butelmann et al., 2022
Patient-specific personalization	Imaging-guided defect-matched printing	PU-gelatin, autologous cell bioinks	Wu et al., 2022; Albanna et al., 2019; Noor et al., 2019

## 4 Tissue-specific opportunities

### 4.1 Osteochondral grafts

Osteochondral grafts serve as a representative example of why AM is a promising adjuvant to TERM. The osteochondral unit is often described as a cartilage and subchondral bone structure, but its function depends on a gradual transition from soft, hydrated, avascular cartilage to mineralized bone. This transition supports load transfer, interface stability, and long-term tissue integration. A construct for osteochondral repair therefore needs to address cartilage formation, support of the bone region, and continuity across the interface (Schaefer et al., 2000). AM offers a means to define the shape of the bone or cartilage tissue *a priori*. It has been shown that osseous tissue of predefined shape can be realized via endochondral ossification of bioprinted mono-material constructs containing high-density mesenchymal stem cells (Gu et al., 2023). From the perspective of bioprinting, the value of AM lies in the spatial organization of cells and bioactive cues rather than mere shaping. Representative studies demonstrate this progression: aspiration-assisted bioprinting assembled chondrogenic and osteogenic spheroids into scaffold-free interfaces, confirming that living microtissues can be spatially orchestrated across tissue regions (Ayan et al., 2020). To recapitulate the stratified layers for cartilage-bone interface reconstruction, two-channel extrusion bioprinting of anisotropic bicellular GelMA/AlgMA hydrogels has been used to spatially place articular cartilage progenitor cells and bone marrow mesenchymal stem cells in order (Zhang Y. et al., 2024). Complementary approaches using dynamic hydrogel bioinks further illustrate that heterogeneous constructs can provide region-specific microenvironments for concurrent regeneration (Dai et al., 2022). Collectively, these studies suggest that clinical relevance demands more than geometric fidelity; constructs must integrate heterogeneous mechanical microenvironments and biological gradients to maintain cartilage phenotype, controlled mineralization, and long-term interfacial integrity.

### 4.2 Skin

Skin is another valuable testbed as it combines stratified architecture with a remodeling barrier, specialized appendages (sweat glands, hair follicles), vascularization, and immune functions. It contains epidermal and dermal compartments, multiple cell populations, extracellular matrix heterogeneity, vascular networks, immune activity, and continuous surface renewal. AM enables the precise organization of dermal matrices and cells to generate layered constructs that match native anatomy. This capacity is pivotal for large full-thickness defected wounds or burns requiring both rapid coverage and controlled tissue organization. *In situ* bioprinting of autologous fibroblasts and keratinocytes accelerated closure of extensive full-thickness wounds in murine and porcine models, demonstrating the ability to conform to patient-specific geometries (Albanna et al., 2019). Moreover, a handheld printer deposited biomaterial and tissue sheets with controlled compositions onto target surfaces, illustrating the practical value of conformal tissue deposition (Hakimi et al., 2018). Complementarily, 3D-bioprinted stabilized and prevascularized skin models and full-thickness skin constructs show that printed skin systems can reproduce layered structure and mimic physiological responses (Kim et al., 2018; Admane et al., 2019). Taken together, these studies confirm that printed skin substitutes must do more than merely recapitulate morphology; they must restore barrier function, support vascularization, and remodel to withstand mechanical and environmental stresses.

### 4.3 Vascular grafts

Vascular grafts represent a third testbed where simple geometry belies complex function. A blood vessel is tubular, but its performance depends on radial organization, circumferential mechanics, compliance, barrier function, endothelial coverage, and resistance to thrombosis. AM contributes by fabricating tubular constructs with controlled wall thickness, channel geometry, and region-specific biological organization. From a bioprinting standpoint, emphasis should be placed on functional conduits rather than shape alone. Microfluidic bioprinting of tough hydrogel-based vascular conduits produced hollow constructs with vein-like and artery-like features, including barrier function, vascular marker expression, and vasomotor responses (Wang D. et al., 2022). Engineered tissue flaps with hierarchical vessel networks show how vessel geometry can be organized across multiple length scales (Szklanny et al., 2021). Printed vessel-like segments using extracellular matrix-derived bioinks reproduced stratified wall organization with tunable mechanics (Potere et al., 2022). These examples illustrate that vascular grafts demand stringent functional fidelity; correct shape is insufficient if compliance, mechanical integrity, or endothelialization are compromised.

Together, these examples illustrate how AM addresses tissue-specific manufacturing challenges in TERM. In each case, bioprinting provides control over graded biological cues, conformal cell delivery, spatial organization, and process reproducibility—features conventional methods struggle to replicate with precision. Crucially, these cases also show that manufacturing control must be intrinsically linked to tissue maturation, host integration, and clinical efficacy. AM achieves its greatest impact when tissue-specific requirements are translated into programmable and testable manufacturing variables rather than being treated as isolated proofs of printability (Ayan et al., 2020; Kim et al., 2018; Potere et al., 2022). Representative studies that aim to provide promising solutions to engineering different tissues with key outcomes are listed in Table 2.

**TABLE 2:** Representative AM studies with quantitative functional benchmarks.

Study	Tissue/Model	AM technique	Key quantitative metric	Functional outcome
Lee et al., 2019	Cardiac/vascular	FRESH extrusion	Feature resolution: ∼20 µm filament; collagen conc.: 6–18 mg/mL	Perfusable cardiac components with anatomical fidelity
Grigoryan et al., 2019	Vascularized tissue	Projection SLA	Channel diameters: ∼1 mm; construct volume: cm^3^ scale	Intravascular mixing and valve-like function
Bernal et al., 2022	Hepatic organoid	Volumetric bioprinting	Print time: seconds; construct scale: Centimeter	Preserved hepatic metabolic function
Han et al., 2021	Cell viability	Extrusion	Shear stress: ∼76–465 Pa (needle flow); DCR: ∼19–38% (43 conditions)	Quantified process-induced cell damage
Wang D. et al., 2022	Vascular conduit	Microfluidic bioprinting	Wall mechanics: Young’s modulus 65.3–472.0 kPa; burst pressure 1,113–1,498 mmHg; vasomotor: Vasoconstriction (PE, 10 µM) + vasodilation (ACh, 10 µM)	Vein-like and artery-like functional features
Kim et al., 2024	Spheroid tissue	High-throughput bioprinting	Throughput: ∼860 spheroids/h (576 in <40 min); spheroid diameter: 300–350 µm	Scalable tissue fabrication
Brown et al., 2021	Osteochondral	Molding + assembly	Interfacial shear strength: 83.0 ± 37.7 kPa (early assembly, 4.9-fold vs. late)	Robust cartilage-bone interface
Freeman et al., 2020	Osteochondral	Growth factor bioprinting	Gradient resolution: 5-Mm core in 8-mm construct; GF dose: VEGF 20–500 ng/mL, BMP-2 200 ng/ml–10 μg/mL	Zone-specific cartilage/bone differentiation

## 5 Limitations of AM in TERM

### 5.1 Printability versus biological functionality

Clinically viable constructs must maintain structural integrity, support tissue-specific cell behavior, and function under physiological loads. However, printability does not equate to biological functionality. Extrusion parameters—including nozzle diameter, pressure, and bioink viscosity—induce shear stress that compromises cell viability, even when macroscopic strand formation appears continuous (Han et al., 2021). While a bioink may exhibit smooth filament formation and stable layer stacking to realize excellent shape fidelity, these properties do not ensure long-term cell survival, tissue maturation, host integration, or functional repair. Current assessments often over-rely on immediate geometry, short-term viability, or early marker expression as primary readouts, rather than demonstrating true regenerative capacity. Complementary network bioinks and dynamic hydrogel bioinks further illustrate this distinction. Although temporary rheological support or dynamic formulation can improve printing behavior, the final construct must ultimately provide a permissive environment for the encapsulated cells to achieve tissue maturation (Hull et al., 2023).

### 5.2 Material limitations and bioink tradeoffs

Material selection remains a fundamental constraint in AM-based TERM. Printable formulations require strict rheological control and rapid sol-gel transitions, requirements that often conflict with cellular needs. Highly printable formulations can be too dense or too stiff to support cell spreading and remodeling. Thus, bioink formulation containing diverse properties through blending could offer a solution. Double network hydrogels offer a means to introduce functional attributes without impacting bioink printability, as illustrated in carboxylated agarose (CA)-collagen hybrid bioinks that yield mechanically stable structures with improved cell adhesion properties without losing print fidelity (Tribukait-Riemenschneider and Shastri, 2025). High-resolution printing has also been demonstrated using a recombinant photocurable collagen-based bioink (Tytgat et al., 2020). Additionally, CA-based bioinks can also be tailored for the mechanical properties and yield free-standing structures under physiological conditions while retaining extrusion conditions that support high cell viability (Forget et al., 2017; Gu et al., 2020). Self-healing bioinks can further improve mechanical attributes of printed structures (Caprioli et al., 2021). Extracellular matrix-reinforced bioinks and *in situ* bioconcrete bioinks demonstrate that material design can improve biological relevance or structural stabilization, but the reliability of each approach still relies on controlled processing and post-printing validation (De Santis et al., 2021; Xie et al., 2022). Conversely, materials mimicking native ECM are often mechanically weak and difficult to process. In sum, these studies underscore the need for multi-objective bioink design balancing printability, mechanics, and bioactivity.

### 5.3 Resolution and scale

In AM, a fundamental resolution-versus-throughput trade-off persists. High-resolution techniques, such as two-photon polymerization, generate micro-scale features but suffer from low throughput and narrow processing windows, limiting clinical scalability (Tytgat et al., 2020). Volumetric bioprinting can fabricate organoid-laden centimeter-scale hydrogel constructs within seconds, but it also requires optically tuned materials, transparent bioresins, and post-printing validation to connect printed architecture with tissue-level metabolic function (Bernal et al., 2022). Self-healing hydrogels printed by digital light processing provide another route toward structured hydrogel fabrication, but material recovery, photochemistry, and construct stability still need to be controlled (Caprioli et al., 2021). This trade-off is important because clinically relevant tissues often require both large volume and microscale organization. A graft may need to match a large, patient-specific defect while also containing local features that guide vascularization, cell migration, or interface formation. Achieving these requirements within a single manufacturing process remains challenging.

### 5.4 Handling and implant stability

Post-printing stability represents a critical yet often overlooked challenge. Constructs must withstand transfer, culture, storage, and implantation without deformation. Soft hydrogel constructs are prone to handling-induced damage, while cell-laden constructs may undergo non-uniform shrinkage during maturation (Machour et al., 2022), indicating that geometric preservation during handling is an engineering problem. After implantation, the construct must resist inflammation, uncontrollable enzymatic degradation, mechanical loading, and limited oxygen supply, while awaiting vascular ingrowth. Evaluations must therefore extend beyond immediate fidelity to include matrix remodeling, phenotypic maintenance, and functional maturation during prolonged culture (Wang M. et al., 2022; Hull et al., 2023; Gu et al., 2023).

### 5.5 Manufacturing, regulatory, and commercialization barriers

Beyond technical performance, AM faces systemic translational barriers. Clinical adoption requires adherence to Good Manufacturing Practice (GMP), including sterile processing, validated raw materials, and defined release criteria. In the regulatory landscape, 3D-bioprinted living tissues are typically classified as combination products (biologic + device) by the FDA and as Advanced Therapy Medicinal Products (ATMPs) by the EMA, requiring interdisciplinary review pathways that are still evolving (Perin et al., 2026; Ricles et al., 2018). The FDA’s 2017 guidance on additive-manufactured medical devices provides a framework for process validation, but it does not extend to biological, cellular, or tissue-based products, leaving living-cell constructs without dedicated guidance on potency, variability, and long-term safety (Adamo et al., 2018; Ricles et al., 2018; US-FDA, 2024). This regulatory gap is compounded by unresolved quality standardization. Bioink quality criteria—encompassing rheological benchmarks, batch-to-batch material specifications, and cell-viability release thresholds—remain conceptually feasible but practically premature: the field has yet to converge on consensus material platforms or validated assay panels (Perin et al., 2026; Schwab et al., 2020), and a multi-laboratory round-robin study has shown that irreducible variability persists even under standardized protocols, complicating quality assurance and technology transfer (Grijalva Garces et al., 2023).

Cell-containing products introduce further complexity regarding cell source, expansion, viability, and phenotype control. Proper storage and transport are also significant when constructs contain living cells or unstable biomolecules. The standardization challenges are not unique to bioprinting. For instance, in FDM-based polymer scaffold fabrication, dimensional accuracy varies with printer type and processing parameters, underscoring the need for standardized reporting of manufacturing conditions (Li et al., 2025). Commercial scalability is further constrained by production costs and time efficiency. Clinical-grade AM setups require capital-intensive equipment, GMP-certified bioink supply chains, and personalized digital workflows whose per-unit cost may exceed that of conventional mass-produced scaffolds. Paradoxically, the high cost of patient-specific imaging, design, and quality control may replace classical manufacturing bottlenecks with economic ones, raising the question of whether personalized AM will remain viable only for high-value indications such as craniofacial or cardiovascular reconstruction. For bioprinted constructs, clinical translation should therefore be discussed through connected workflows that include biological material preparation, printing, maturation, sterility control, functional testing, and delivery to the target defect. Patient-specific cardiac bioprinting and *in situ* skin bioprinting show that personalization becomes translationally meaningful only when anatomical matching is paired with robust biological performance and scalable logistics (Noor et al., 2019; Albanna et al., 2019).

Overall, the principal limitation of AM lies in translating structural control into durable biological function - a challenge compounded by the need to embed this technical element within compliant, scalable, and economically viable manufacturing. The field must transition from demonstrating printability to proving maturation, integration, and compliance with standardized performance requirements. Thus, the key translational barriers include evolving regulatory pathways for living-cell constructs, the absence of bioink quality standards, irreducible manufacturing variability, cell sourcing and phenotype control, cold-chain logistics, and the economic paradox of personalization. Addressing these is a prerequisite to establishing AM as a reliable manufacturing strategy for TERM, rather than merely a powerful laboratory tool.

## 6 What the field needs next

For AM to have lasting impact in TERM, it must transition from structural demonstration to rigorous functional validation. Printed constructs should be engineered as dynamic microenvironments capable of orchestrating cell survival, matrix deposition, vascular ingrowth, immune modulation, and tissue-specific maturation (Figure 2). Critically, these advances must not compromise scalability. To support this transition, the field requires standardized reporting frameworks for bioink composition, printing parameters, construct fidelity, mechanical properties, cell outcomes, and maturation protocols. Functional endpoints should be selected based on the target tissue physiological functions rather than solely relying on generic viability or morphological metrics. From a translational perspective, future bioink development should also balance printability, biological function, reproducibility, and clinical feasibility, rather than optimizing printing behavior alone (Gu et al., 2022).

**FIGURE 2:**
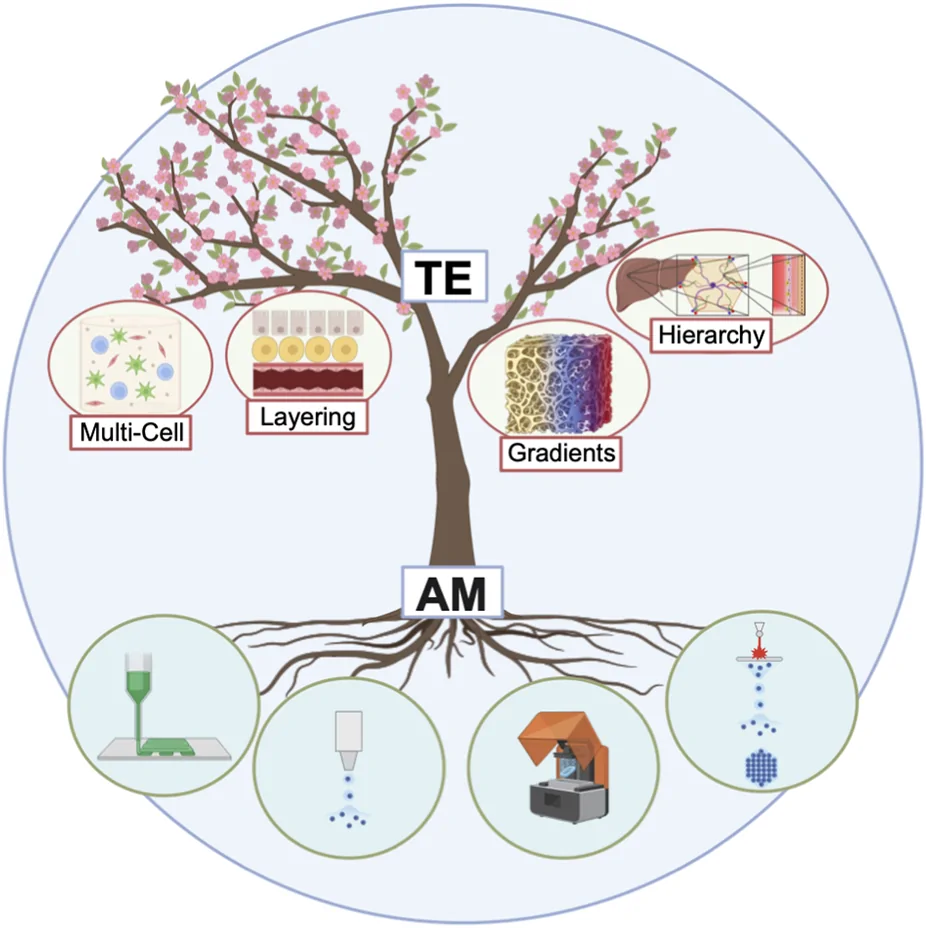
Schematic of the future of tissue engineering (TE) integrated with the ecosystem (soil) of additive manufacturing techniques (L-R: direct ink writing (DIW), commonly referred to as extrusion bioprinting, drop-on-demand (DOD) printing, stereolithography printing (STL), digital light processing (DLP)) nourishing the development of solutions that build-off classical TE strategies, yielding innovative breakthroughs in structured tissues with layered and hierarchical cellular and biomaterial architecture, incorporating biological and structural gradients, supporting the clinical translation of TERM. Created in BioRender.

Future manufacturing workflows should integrate imaging, computational modeling, artificial intelligence-assisted design, and quality control to establish closed-loop feedback systems that couple architectural design with biological performance. Machine learning is now reshaping this pipeline from material formulation through process optimization to real-time control (Zhang et al., 2025). At the material level, data-driven approaches enable *de novo* design of hydrogel formulations by mining structure–property relationships from experimental datasets and iteratively optimizing composition, substantially reducing the trial-and-error experimentation that currently dominates bioink development (Liao et al., 2025; Zhang et al., 2025). Machine-learning models can further correlate bioink rheology, printing parameters, and post-printing construct fidelity to predict printability *a priori* (Nadernezhad and Groll, 2022; Ruberu et al., 2021), and have been extended to forecast post-printing cell viability from process parameters, directly linking manufacturing decisions to biological outcomes (Zhang et al., 2024a). On the quality-control side, deep-learning-based monitoring systems can detect extrusion defects in real time and automatically adjust printing parameters, moving bioprinting from open-loop deposition toward autonomous, self-correcting fabrication (Bonatti et al., 2022; Sani et al., 2024).

Looking beyond current AM paradigms, 4D printing—incorporating stimuli-responsive materials that undergo programmed shape transformation post-fabrication—offers a complementary route to dynamic, minimally invasive tissue delivery and on-demand functional adaptation (Mirasadi et al., 2026; Yarali et al., 2024). Integrating such responsive platforms with AM-based TERM workflows could enable constructs that actively remodel in response to physiological cues.

## 7 Conclusion

AM is not a universal solution for TERM’s challenges, but it provides a robust manufacturing framework for tackling prevailing bottlenecks in construct architecture, spatial patterning, process consistency, patient-specific design, and clinical translation. Its impact will depend less on the capacity to fabricate geometrically complex constructs and more on the ability to engineer systems that support tissue maturation, withstand clinical handling, fulfill functional demands, and integrate into viable clinical workflows. Future progress will therefore require closer integration between digital design, biomaterial selection, cell biology, quality control, and regulatory planning.
